# Employment of Hydrastinin in Uterine Hemorrhages

**Published:** 1892-09

**Authors:** 


EMPLOYMENT OF HYDRASTININ IN UTERINE HEMORRHAGES.—(*Archives of Gynecology*, May, 1892)—Dr. Emanuel reports the use of hydrastinin hydrochlorate in forty-eight cases in Czempin's clinic in Berlin, three-eighths of a grain being given in gelatin capsules three or four times a day after the hemorrhage had begun, with the following results: twenty-six of the forty-eight were so influenced by the remedy that the bleeding ceased in the succeeding twenty-four to thirty-six hours, a result which has not been obtained by any other known remedies. Hydrastinin seems to act on the small vessels of the mucosa, whereas ergot exerts its influence upon the smooth muscular fibre. Hence the former does not take the place of ergot in *post-partum* hemorrhages or hemorrhages after abortion. No disagreeable symptoms were observed from the employment of hydrastinin.—*Ex.*

